# Four Different Methods Comparison for Extraction of Astaxanthin from Green Alga *Haematococcus pluvialis*

**DOI:** 10.1155/2014/694305

**Published:** 2014-01-19

**Authors:** Shengzhao Dong, Yi Huang, Rui Zhang, Shihui Wang, Yun Liu

**Affiliations:** Beijing Key Laboratory of Bioprocess, The Biorefinery Research and Engineering Center of the Ministry of Education of China, College of Life Science and Technology, Beijing University of Chemical Technology, Beijing 100029, China

## Abstract

*Haematococcus pluvialis* is one of the potent organisms for production of astaxanthin. Up to now, no efficient method has been achieved due to its thick cell wall hindering solvent extraction of astaxanthin. In this study, four different methods, hydrochloric acid pretreatment followed by acetone extraction (HCl-ACE), hexane/isopropanol (6 : 4, v/v) mixture solvents extraction (HEX-IPA), methanol extraction followed by acetone extraction (MET-ACE, 2-step extraction), and soy-oil extraction, were intensively evaluated for extraction of astaxanthin from *H. pluvialis*. Results showed that HCl-ACE method could obtain the highest oil yield (33.3 ± 1.1%) and astaxanthin content (19.8 ± 1.1%). Quantitative NMR analysis provided the fatty acid chain profiles of total lipid extracts. In all cases, oleyl chains were predominant, and high amounts of polyunsaturated fatty acid chains were observed and the major fatty acid components were oleic acid (13–35%), linoleic acid (37–43%), linolenic acid (20–31%), and total saturated acid (17–28%). DPPH radical scavenging activity of extract obtained by HCl-ACE was 73.2 ± 1.0%, which is the highest amongst the four methods. The reducing power of extract obtained by four extraction methods was also examined. It was concluded that the proposed extraction method of HCl-ACE in this work allowed efficient astaxanthin extractability with high antioxidant properties.

## 1. Introduction

Astaxanthin, one of the main xanthophyll carotenoid pigments, possesses 500-fold and 38-fold times stronger free radical antioxidant activity of vitamin E and *β*-carotene, respectively [1]. Owing to its strong antioxidant properties, astaxanthin plays an important role in protection against inflammation, UV-light photooxidation, aging and age-related macular degeneration, and cancer and in enhancement of the immune response, liver function, heart health, and so forth [2]. Hence, astaxanthin has widespread applications in the pharmaceutical, cosmetic, food, and feed industries [3]. Nowadays, astaxanthin has two main commercially available formations: chemical synthesis and natural resources from microalgae, yeast, and crustacean byproducts. Though dominating the current world market, chemical synthetic astaxanthin has been banned into health food market by the U.S. Food and Drug Administration (FDA) due to its low bioavailability and security. Therefore, astaxanthin obtained from natural resources has triggered more and more attention in recent years [4].

As one of the potent organisms for production of astaxanthin,* Haematococcus pluvialis* accumulates high content of natural astaxanthin up to 9.2 mg/g cell [5]. From this viewpoint, it is a challenge for us to extract efficiently the astaxanthin from *H. pluvialis* cell using several strategies. Sarada et al. [1] evaluated extractability of astaxanthin from cyst cells by treating cells with various solvents and pretreating the cells with organic and mineral acids at 70°C. It was shown that hydrochloric acid treatment facilitated 86–94% extractability of astaxanthin. Kobayashi et al. [6] treated *H. pluvialis* cells with 40% (v/v) acetone for 2 min at 80°C, followed by lyophilization or treatment of cells with specific lytic enzymes. By these treatments, the extractability of the astaxanthin achieves 70%. In et al. [7] extracted the astaxanthin by treating *H. pluvialis* cells with several enzymes, and the maximal extractability is 2649 ± 359 *μ*g/g cell. Kang and Sim [8] treated *H. pluvialis* cells with common vegetable oils and the astaxanthin oil yields reach more than 88%. Although several reports on extraction of astaxanthin from *H. pluvialis* have been available; however, no efficient method has been achieved due to the thick cell wall of organism hindering solvent extraction of astaxanthin.

In this study, four different methods, hydrochloric acid pretreatment followed by acetone extraction (HCl-ACE), hexane/isopropanol (6 : 4, v/v) mixture solvents extraction (HEX-IPA), methanol extraction followed by acetone extraction (MET-ACE, 2-step extraction), and soy-oil extraction, were employed to extract astaxanthin from *H. pluvialis*. The aims of this work are focused on (1) comparing and screening a suitable proposed method for extraction of astaxanthin from *H. pluvialis* organism; (2) elucidating the fatty acid chain profiles of total lipid extracts in all cases using NMR; (3) evaluating the antioxidant properties of lipid extracts in terms of DPPH radical scavenging activity and reducing power capacity.

## 2. Materials and Methods

### 2.1. Materials

*Haematococcus pluvialis *organisms were gifted by Yunnan Yunlin Biological Technology Co. Ltd. (Kunming, Yunnan province, China). Before extraction, the organism was dried and the moisture content was below 0.5 wt.%. Soy oil was brought from Jinhai Food Industry Co. Ltd. (Qinhuangdao, China). Astaxanthin standard with purity of 99% was purchased from Sigma (St. Louis, MO). All other chemicals were of analytic grade and bought from local market.

### 2.2. Extraction Techniques

#### 2.2.1. HCl-ACE Extraction

Astaxanthin extraction by HCl-ACE method was modified according to the procedures reported by Sarada et al. [1] HCl-ACE extraction procedures included two steps; firstly, ten milligrams lyophilized biomass was treated with 1 mL of 4 M HCl in a centrifugal tube at 70°C for 2 min. The sample was cooled and centrifuged at 5000 rpm for 5 min. Then, the HCl-treated sample was washed twice by distilled water and resuspended in 1 mL acetone. The mixture was ultrasonically extracted in an ice-water bath for 20 min and then centrifuged at 3500 rpm at 4°C for 6 min. The supernatants were used for HPLC estimation of extractable astaxanthin. All the steps were carried out in light protection and filled with nitrogen.

#### 2.2.2. HEX-IPA Binary Solvents Extraction

HEX-IPA binary solvents extraction method consists of transferring 10 mg of the lyophilized organisms into 2 mL of hexane/isopropanol (6 : 4, v/v) binary organic solvents for 20 min in an ice-water bath temperature and ultrasonically assistant extraction. The mixture of cell biomass, extract, and solvent was separated by means of centrifugation at 3500 rpm at 4°C for 5 min, followed by concentration under vacuum. The extraction yield was calculated in dry basis and expressed in % (w/w-dry basis). HPLC estimation was employed for analysis of astaxanthin content. All the steps were carried out in light protection and filled with nitrogen.

#### 2.2.3. MET-ACE 2-Step Extraction

In this procedure, ten milligrams biomass was weighed into a 15 mL screw top amber glass vial and ultrasonically extracted in an ice-water bath with 1 mL methanol and acetone for 5 min in sequential order [10]. For first extraction step, the sample was extracted with 1 mL methanol in 15 mL screw top amber glass vial and centrifuged at 3500 rpm at 4°C for 5 min. Then 1 mL acetone was added to glass vial and extraction for second step. Extracts were combined for 2-step extraction and used for HPLC estimation of extractable astaxanthin. All the steps were carried out in light protection and filled with nitrogen.

#### 2.2.4. Oil-Soy Extraction

The oil-soy extraction method was performed in triplicate according to the procedure presented by Sachindra and Mahendrakar [11]. The extraction yield was evaluated using soybean oil as solvent. The method consisted of mixing 2.5 g cell biomass with 20 mL vegetable oil in a 250 mL flask (light protected), submitted to hot plates with 2 h agitation period at room temperature. Further, the oil extracts were recovered by cellulose filtration (0.22 *μ*m) and the extraction yield and content of astaxanthin was quantified by HPLC analysis.

### 2.3. HPLC Determination of Astaxanthin and Evaluation of Extract Quality

#### 2.3.1. Extraction Yield

Oil consisting of astaxanthin extraction yield by different methods was calculated with

(1) $$\begin{array}{c} \text{Extraction}\;\text{yield}=\frac{W_{\text{oil}}}{W_{\text{raw}\;\text{material}}}\times 100\%, \end{array}$$

where *W*_oil_ is the oil weight (*μ*g) obtained by different method after concentration under vacuum; *W*_raw  material_ is the mass (mg) of *H. pluvialis *organisms. All trials were carried out in triplicate.

#### 2.3.2. SEM for Morphology of* H. pluvialis* Cell

To evaluate the extraction efficiency of different methods, the morphology of *H. pluvialis *cells before and after extraction was recorded by a scanning electron microscope (SEM) (SU1510; Hitachi, Hitachi City, Japan). Before SEM analysis, the sample was washed gently with 50 mmol/L phosphate buffer (pH 7.2) and fixed with 100 mL glutaraldehyde (2.5%) and 100 mL osmic acid solution (1%). The specimen was dehydrated using sequential ethanol and tertiary butyl alcohol. After dehydration, the specimen was dried with carbon dioxide (CO_2_) and sputter-coated with gold in an ion coater for 2 min [12].

#### 2.3.3. HPLC Analysis for Astaxanthin Content

The extracts were subjected to high performance liquid chromatography (HPLC) (LC-20AT; Shimadzu, Beijing, China) equipped with ZORBOX 300-SB C18 column for astaxanthin content determination. The conditions were as follows: eluants were (A) acetone and (B) methanol: H_2_O (9 : 1 v/v) with the flow rate of 0.8 mL/min and column temperature was 40°C. A gradient concentration program was employed as follows: B was run at 80 to 20% for 25 min, 20% for 10 min, and 20 to 80% for 5 min. The detection wavelength was monitored at 460 nm.

#### 2.3.4. ^1^H-NMR for Fatty Acids Profiles

The fatty acid profiles in lipid extracts were quantitatively analyzed by ^1^H-NMR method, which is based on the fact that the amplitude of ^1^H-NMR signal is proportional to the number of hydrogen nuclei contained in the molecule [13]. For ^1^H-NMR, 10 mg of the sample was dissolved in 0.6 mL of CDCl_3_ and the spectrum was recorded at 25°C on a Bruker Avance II 600 MHz spectrometer (Bruker Daltonics, Billerica, MA, USA); 30 scans for each sample were taken during the measurement. A standard 4 mm quadronuclei (^1^H) probe (QNP) was used. An acquisition time of 3.9 s, a relaxation delay of 1 s, a flip angle of 30°, and a sweep width of 4.139 kHz were employed in the spectral measurements. TMS with the concentration of 0.03% (v/v) was used as an internal standard. The fatty acid composition was calculated as follows:

(2) $$\begin{array}{c} \text{Linolenic}\;\left(\text{Ln}\right)\%=\left[\frac{B}{A+B}\right]\times 100\%,\\ \text{Linoleic}\;\left(\text{L}\right)\%=\left[\frac{E}{D}-2\times \text{Ln}\%\right]\times 100\%,\\ \text{Oleic}\;\left(\text{O}\right)\%=\left[\frac{C}{2}\times D-\text{l}\%-\text{Ln}\%\right]\times 100\%,\\ \text{Saturated}\;\text{FA}\;\%=\left[1-\left(\frac{C}{2}\times D\right)\right]\times 100\%, \end{array}$$

where *A*, *B*, *C*, *D*, and *E* are the areas of the peaks with chemical shift ranges as listed in Table 1.

#### 2.3.5. DPPH (1,1-Diphenyl-2-picrylhydrazyl) Radical Scavenging Activity

The antioxidant activity of the extracts was measured on the basis of the scavenging activity of the stable DPPH free radical [14]. A volume of 1.5 mL of each sample was added to 1.5 mL of 0.1 mmol/L DPPH in ethanol. The mixture was slightly shaken and allowed to stand for 30 min at room temperature in darkness. The absorbance of the resulting solution was measured at 517 nm in a UV756CRT spectrophotometer (Shanghai Youke Instrument Co. Ltd., Shanghai, China). Therefore the DPPH radical scavenging activity can be obtained by the following equation:

(3) DPPH  radical  scavenging  activity  =(1−Ai−AjA0)×100%,

where *A*_0_ and *A*_*i*_ are the absorbance of DPPH at 517 nm in the absence and presence of sample, respectively. *A*_*j*_ is the absorbance of sample alone.

#### 2.3.6. Test for Reducing Power

Each sample of 1 mL was added to 2.5 mL of 0.2 mol/L phosphate buffers (pH 6.6) and 1 mL 1% (w/v) potassium ferricyanide. The mixture was incubated at 50°C for 20 min and cooled rapidly. Then 2.5 mL of 10% (w/v) trichloroacetic acid was added to the mixture, which was then centrifuged at 3500 rpm for 10 min. The supernatant (2.5 mL) was mixed with 2.5 mL of distilled water and 0.5 mL of 0.1% (w/v) ferric chloride in a test tube. After a 10 min reaction, the absorbance of the resulting solution was measured at 700 nm by a UV756CRT spectrophotometer [15].

### 2.4. Statistical Analysis

All reported data were collected in triplicate, and the statistical analysis was performed using SAS 9.0 software (SAS Institute, Inc., Cary, NC, USA). Analytical data were expressed as mean ± SE (standard error of the mean).

## 3. Results and Discussion

### 3.1. Effect of Different Extraction Methods on Oil Yield and Astaxanthin Content

The extraction yield and total astaxanthin content (TAC) values obtained by four different extraction techniques are presented in Table 1, together with the solvent/solid ratio and the extraction time. Evaluating the results from Table 2, it was observed that HCl-ACE extraction method presented the highest extraction oil yield and TAC with the values of 33.3 ± 1.1% (w/w) and 19.8 ± 1.1 mg/g-cell, respectively. Although HEX-IPA and MET-ACE methods showed the similar extraction oil yield, the latter possessed a higher TAC extraction. In view of oil-soy method, the second extraction oil yield and TAC with the values of 26.0 ± 1.0% (w/w) and 0.9 ± 0.1 mg/g-cell, respectively. Improved carotenoid extraction has been demonstrated previously when biomass was pretreated [16]. And Scaife et al. [10] demonstrated that there was a conditional synergistic improvement in extraction efficiency. For this, the solvents must be employed sequentially, in a predefined order. Synergy, with respect to carotenoid extraction, has been reported previously, when a liquid nitrogen and dimethyl sulphoxide pretreatment combined with acetone: methanol (7 : 3) extraction solvent significantly increased extraction efficiencies [17]. However, these synergy phenomena were not observed in our work; the reasons were probably that methanol or hexane extraction weakens the interaction of astaxanthin with the biological material or increases the permeability of the biomass, leaving the astaxanthin prone to solvation by acetone or isopropane.

**Figure 1:**
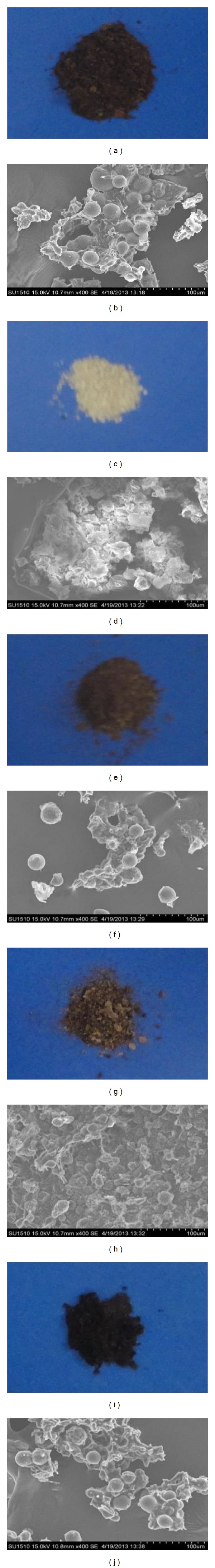
Photoes and SEM analysis of *H. pluvialis* cell after different extraction methods. (a) *H. pluvialis* cell material photo before extraction, (b) cell SEM before extraction; (c) *H. pluvialis* cell material photo after HCI-ACE extraction, (d) cell SEM after HCI-ACE extraction; (e) cell material photo after HEX-IPA extraction, (f) cell SEM after NEX-IPA extraction; (g) cell material photo after MET-ACE extraction, (h) cell SEM after MET-ACE extraction; (i) cell material photo after oil-soy extraction, (j) cell SEM after oil-soy extraction.

The morphologies of *H. pluvialis *cells before and after extraction were detected by SEM and the photographs are shown in Figure 1. As can be seen in Figures 1(a) and 1(b), the untreated cells are dark brown and intact with no signs of pitting or damage to the cell wall. After the treatment of HCl-ACE, the cells become almost white (Figure 1(c)) and the cell wall is damaged and wrinkled strongly (Figure 1(d)), indicating that a majority of astaxanthin in the cells was extracted. The morphologies of the cells treated by HEX-IPA and MET-ACE show similar properties, that is, brown color (Figures 1(e) and 1(g)) and moderate damage of cell wall (Figures 1(f) and 1(h)). The color of the cells treated by oil-soy is almost the same with the untreated cells (Figures 1(a) and 1(i)), and the cell wall is just slightly changed (Figures 1(b) and 1(j)). These results are in agreement with the extraction oil yields and TACs listed in Table 2.

### 3.2. Effect of Different Extraction Methods on Fatty Acid Profiles in Extracts by NMR

**Figure 2:**
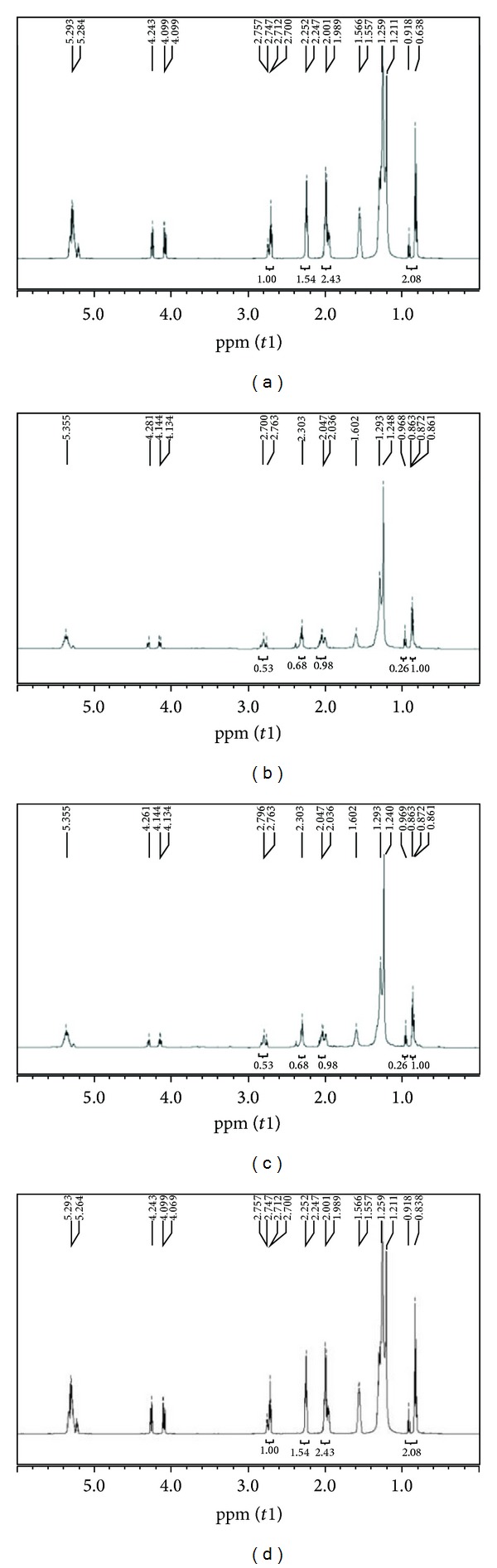
NMR determination for fatty acids profiles of extracts obtained by four different extraction techniques from *H. pluvialis*. ((a) NMR analysis of extract by HCI-ACE method; (b) NMR analysis of extract by HEX-IPA method; (c) NMR analysis of extract by MET-ACE method; (d) NMR analysis of extract by oil-soy method.)

^1^H-NMR spectroscopy experiments were carried out in order to gather information about the quantitative fatty acid (FA) composition in the total lipid extract by four different extraction methods and the results are deposited in Figure 2. As the area of the signals in the ^1^H NMR spectra is proportional to the number of hydrogens of each type in the sample, the fatty acid composition can be determined through the relation between the areas from the characteristic signals of each fatty acyl chain and one of those from the glycerol backbone in the ^1^H NMR spectra. The FA compositions of lipid extract by four extraction methods are shown in Table 3. It can be seen from Table 3 that FA profiles in extract obtained by HCl-ACT, HEX-IPA, MET-AC, and oil-soy methods are similar, but the percentage of each FA content is different. In conclusions the effect of extraction methods on FA profiles in total lipid extracts was greatly significant in its content. It was reported, that, in rats, a low dietary ratio of (*n* − 6)/(*n* − 3) PUFA (poly unsaturated fatty acid) induces an increase in the relative concentration of *n* − 3 PUFA in bone and caused a reduction in prostaglandin E2 (PGE2) and an increase in serum bone alkaline phosphatase [18]. Recently, two large human epidemiological studies have found that higher (*n* − 6)/(*n* − 3) PUFA ratio and higher saturated FA in the diet are associated with lower bone mineral density [19].

### 3.3. Effect of Four Different Methods on Antioxidation of Extracts

**Figure 3:**
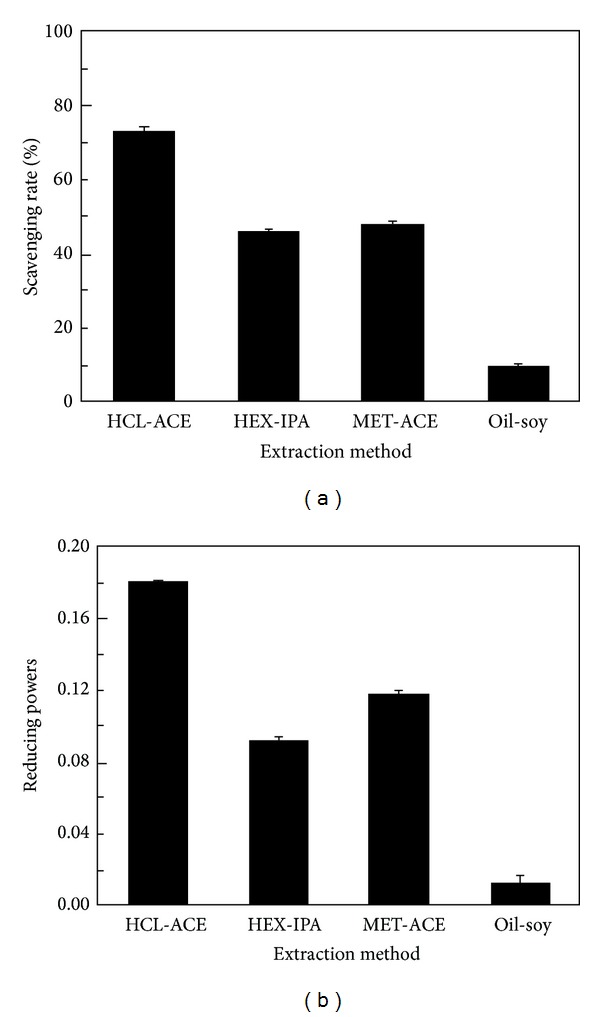
DPPH radical scavenging activities (a) and reducing powers (b) of astaxantin products from *H. pluvialis* extracted by HCI-ACE, HEX-IPA, MET-ACE, and oil-soy methods.

To evaluate the effect of four different extraction methods on the quality of total lipid extracts from *H. pluvialis*, DPPH free radical-scavenging assay and reducing power experiments were carried out in this work and the results are deposited in Figure 3. It was indicated that scavenging activities and reducing power of extract obtained by HCl-ACE extraction method were the highest, respectively. While scavenging activities and reducing power of extract obtained by oil-soy extraction method were the lowest, the reasonable explanation was probably that the astaxanthin content in extract obtained by HCl-ACE was the highest, while the astaxanthin content in extract obtained by oil-soy was the lowest. It suggested that astaxanthin was the prominent factor for the antioxidant character of extracts obtained by four extraction methods. The antioxidant effect of astaxanthin content agreed with the previous reports [20].

## 4. Conclusions

The total oil yield and TCA extraction from *H. pluvialis* by four different techniques presented a strongly significant difference, which are technically viable depending on the astaxanthin content of extracts. The best extraction method, in terms of oil yield, TCA, and antioxidant of extract, was HCl-ACE procedure. SEM, NMR, and DPPH assay and reducing power experiments were carried out to further confirm that HCl-ACE procedure was suitable for extraction of asatxanthin from *H. pluvialis* biomass.

## Figures and Tables

**Table 1:** ^
1^H-NMR spectral peak assignment*.

Signal	Chemical shift (ppm)	Functional group
1	0.82–0.94	–CH_3_ (terminal methyl protons (saturated, oleic and linoleic))
2	0.94–1.03^a^	–CH_3_ (terminal methyl protons (linolenic))
3	1.20–1.43	–(CH_2_)n–(methylene protons (saturated))
4	1.55–1.69	–OCO–CH_2_–CH_2_–(_–methylene protons (carbonyl))
5	1.93–2.13	–CH_2_–CH CH–(allyl methylene protons)
6	2.25–2.36	–OCO–CH_2_–(_–methylene protons)
7	2.73–2.87	HC–CH_2_–CH (divinyl methylene protons)
8	4.10–4.35	–CH_2_OCOR (methylene protons (glyceryl))
9	5.23–5.29	CHOCOR (proton on carbon atom 2 of glyceryl group)
10	5.29–5.43	–CH CH–(olefinic protons)

*Chemical shift ranges shown were adapted from published data [9] and these values were used as integration limits for measurement of peak areas.

^a^This chemical shift range was changed to 0.94–0.99 ppm in all subsequent peak integration measurements to exclude signal contribution from unassigned peak at 1.01 ppm.

**Table 2:** Effect of different extraction methods on oil yield and total astaxanthin content (TAC) of extracts from *H. Pluvialis* cells.

Extraction technique^a^	Solvent/raw material ratio (mL/g)	Extraction time (min)	Extraction oil yield (%, w/w)	TAC (mg/g-cell)
HCl-ACE	200	20	33.3 ± 1.1	19.8 ± 1.1
HEX-IPA	100	20	23.7 ± 2.3	9.7 ± 0.6
MET-ACE	400	20	24.3 ± 0.6	13.8 ± 0.4
Oil soy	8	120	26.0 ± 1.0	0.9 ± 0.1

^a^HCl: hydrochloric acid; ACE: acetone; HEX: hexane; IPA: isopropanol; MET: methanol; HCl-ACE: hydrochloric acid + acetone (5 : 5); Hex-IPA: hexane + isopropanol (6 : 4); MET-ACE: methanol + acetone (5 : 5); oil soy: extraction with soybean oil.

**Table 3:** ^
1^H-NMR spectroscopy results for FA compositions profiles*.

Fatty acid compositions (%)	HCl-ACE	HEX-IPA	MET-ACE	Oil-soy
Linolenic acid (*n* − 3)	25.93	20.75	20.63	30.8
Linoleic acid (*n* − 6)	42.81	17.89	36.67	34.9
Oleic acid (monounsaturated FA)	13.93	34.54	14.75	14.0
Saturated FA	17.33	26.82	27.94	21.1
(*n* − 6)/(*n* − 3)	1.65	0.86	1.78	1.13
Total monounsaturated FA/total Saturated FA	0.80	1.29	0.53	0.66
Total PUFA/total Saturated FA	3.97	1.44	2.05	3.11

*HCl: hydrochloric acid; ACE: acetone; HEX: hexane; IPA: isopropanol; MET: methanol; HCl-ACE: hydrochloric acid + acetone (5 : 5); Hex-IPA: hexane + isopropanol (6 : 4); MET-ACE: methanol + acetone (5 : 5); oil soy: extraction with soybean oil.

## References

[1] Sarada R, Vidhyavathi R, Usha D, Ravishankar GA (2006). An efficient method for extraction of astaxanthin from green alga *Haematococcus pluvialis*. *Journal of Agricultural and Food Chemistry*.

[2] Guerin M, Huntley ME, Olaizola M (2003). *Haematococcus astaxanthin*: applications for human health and nutrition. *Trends in Biotechnology*.

[3] De Holanda HD, Netto FM (2006). Recovery of components from shrimp (*Xiphopenaeus kroyeri*) processing waste by enzymatic hydrolysis. *Journal of Food Science*.

[4] Seabra LMJ, Pedrosa LFC (2010). Astaxanthin: structural and functional aspects. *Revista de Nutricao*.

[5] Domínguez-Bocanegra AR, Ponce-Noyola T, Torres-Muñoz JA (2007). Astaxanthin production by *Phaffia rhodozyma* and *Haematococcus pluvialis*: a comparative study. *Applied Microbiology and Biotechnology*.

[6] Kobayashi M, Kurimura Y, Sakamoto Y, Tsuji Y (1997). Selective extraction of astaxanthin and chlorophyll from the green alga *Haematococcus pluvialis*. *Biotechnology Techniques*.

[7] In MJ, Choi JH, Kim S, Chae HJ, Kim DH (2008). Enhanced Extraction of astaxanthin from *Haematococcus pluvialis* using enzyme treatments. *Journal of the Korean Society for Applied Biological Chemistry*.

[8] Kang CD, Sim SJ (2008). Direct extraction of astaxanthin from *Haematococcus* culture using vegetable oils. *Biotechnology Letters*.

[9] Guillén MD, Ruiz A (2003). Rapid simultaneous determination by proton NMR of unsaturation and composition of acyl groups in vegetable oils. *European Journal of Lipid Science and Technology*.

[10] Scaife MA, Ma CA, Armenta RE (2012). Efficient extraction of canthaxanthin from *Escherichia coli* by a 2-step process with organic solvents. *Bioresource Technology*.

[11] Sachindra NM, Mahendrakar NS (2005). Process optimization for extraction of carotenoids from shrimp waste with vegetable oils. *Bioresource Technology*.

[12] Kockro RA, Hampl JA, Jansen B (2000). Use of scanning electron microscopy to investigate the prophylactic efficacy of rifampin-impregnated CSF shunt catheters. *Journal of Medical Microbiology*.

[13] Liu Y, Wu H, Yan Y, Dong L, Zhu M, Liang P (2013). Lipase-catalyzed transesterification for biodiesel production in ionic liquid [Emim]Tfo. *International Journal of Green Energy*.

[14] Sun L, Lee HK (2003). Optimization of microwave-assisted extraction and supercritical fluid extraction of carbamate pesticides in soil by experimental design methodology. *Journal of Chromatography A*.

[15] Yuan C, Du L, Jin Z, Xu X (2012). Storage stability and antioxidant activity of complex of astaxanthin with hydroxypropyl-beta-cyclodextrin. *Carbohydrate Polymers*.

[16] Macías-Sánchez MD, Mantell C, Rodríguez M, Martínez de la Ossa E, Lubián LM, Montero O (2009). Comparison of supercritical fluid and ultrasound-assisted extraction of carotenoids and chlorophyll a from *Dunaliella salina*. *Talanta*.

[17] Valduga E, Valério A, Tatsch PO, Treichel H, Furigo A, Di Luccio M (2009). Assessment of cell disruption and carotenoids extraction from *Sporidiobolus salmonicolor* (CBS 2636). *Food and Bioprocess Technology*.

[18] Reinwald S, Li Y, Moriguchi T, Salem N, Watkins BA (2004). Repletion with (n-3) fatty acids reverses bone structural deficits in (n-3)-deficient rats. *Journal of Nutrition*.

[19] Corwin RL, Hartman TJ, Maczuga SA, Graubard BI (2006). Dietary saturated fat intake is inversely associated with bone density in humans: analysis of NHANES III. *Journal of Nutrition*.

[20] Chen X, Chen R, Guo Z, Li C, Li P (2007). The preparation and stability of the inclusion complex of astaxanthin with *β*-cyclodextrin. *Food Chemistry*.

